# Segmentation of toddler nutritional status using REBUS and FIMIX partial least square in Southeast Sulawesi

**DOI:** 10.1016/j.mex.2023.102515

**Published:** 2023-12-12

**Authors:** Bambang Widjanarko Otok, Purhadi, Riry Sriningsih, Dalbergia Septi Dila

**Affiliations:** a Department of Statistics, Faculty of Science and Data Analytics, Institut Teknologi Sepuluh Nopember, Surabaya 60111, Indonesia; b Department of Mathematics, Faculty of Mathematics and Natural Sciences, Universitas Negeri Padang, West Sumatera, Indonesia

**Keywords:** FIMIX-PLS, Nutrition status, REBUS-PLS, SEM-PLS, Southeast Sulawesi, Toddlers, REBUS-PLS and FIMIX-PLS

## Abstract

Nutrition is one of the important factors that play a major role in the growth and development of children so that they can develop optimally. Child malnutrition, such as stunting, underweight, and wasting, is a significant problem in Indonesia. The World Health Organization (WHO) determined that the nutritional status of children under five in Indonesia is in the chronic category, one of which is in Southeast Sulawesi Province. This study examines and analyzes the factors that influence the nutritional status of children under five in Southeast Sulawesi using the Partial Least Square Structural Equation Modeling (SEM-PLS) method and then segments the nutritional status of children under five using Response Based Unit Segmentation Modeling in Partial Least Square (REBUS-PLS) and Finite Mixture Partial Least Square (FIMIX-PLS). The number of observations in this study was 216 sub-districts. From the results of the SEM-PLS analysis conducted, it was concluded that the 10 indicators used were valid and significant in describing the latent variables, and the practice factor variable had an effect on the food factor variable, the food factor variable had an effect on the service factor variable, and the service factor variable had an effect on the under-five nutritional status variable. The REBUS-PLS analysis results in two segments, with one segment of 75 observations and the other segment of 141 observations. The same conclusion is obtained as in the SEM-PLS analysis, but the results of the analysis with REBUS-PLS have a greater value than the results of the SEM-PLS analysis. Key points of the article:

1. Comparing REBUS-PLS and FIMIX-PLS methods for overcoming the case of heterogeneity in dat.
2. Combining the SEM-PLS method with the REBUS and FIMIX methods in discussing the factors that influence the nutritional status of children under five in Southeast Sulawesi.

## Graphical abstract

**Figure:**
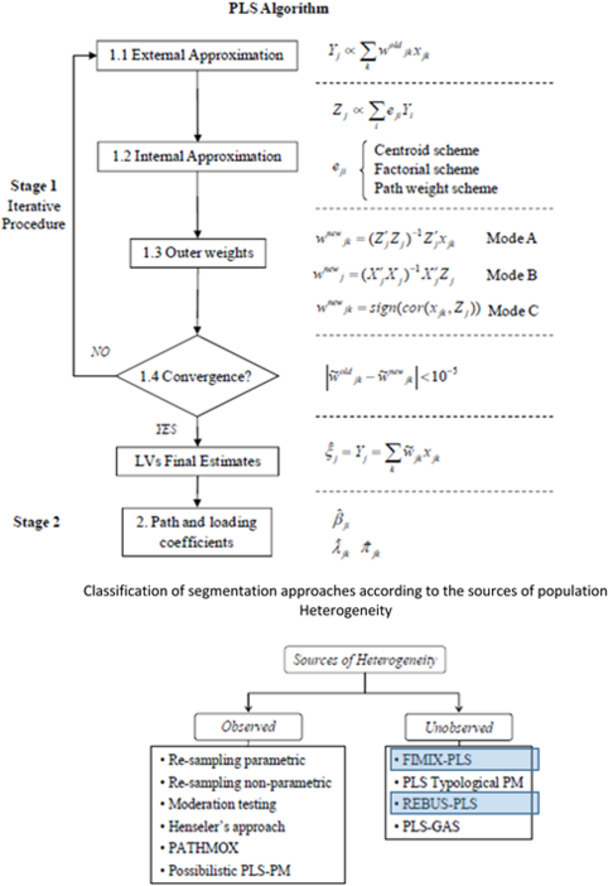

Specifications table

**Table:** 

Subject area:	Mathematics and Statistics
More specific subject area:	*Statistics: Non-parametric modeling*
Name of your method:	*REBUS-PLS and FIMIX-PLS*
Name and reference of original method:	M. A. Mukid, B. W. Otok, and A. Suharsono, “Segmentation in Structural Equation Modeling Using a Combination of Partial Least Squares and Modified Fuzzy Clustering,” Symmetry (Basel)., vol. 14, no. 11, 2022.
Resource availability:	*Southeast Sulawesi Nutrition Status Concept*

## Method details

### Introduction

Nutrition is an important factor that must be considered during the growth period of children. The growth period of children is very closely related. Providing insufficient nutritional intake in the womb will adversely affect the growth of children in the future, and can even cause death. In addition to causing death, malnutrition can also cause children to experience several growth problems such as stunting, underweight and wasting. Stunting is a condition of failure to thrive in infants under five years old (toddlers) due to chronic malnutrition so they have a shorter height than their age. Underweight is a condition where children under five years old weigh less than their peers due to malnutrition. Meanwhile, wasting is a growth disorder where children have less weight for height than their peers. Malnourished toddlers will be more susceptible to disease and have a level of intelligence that is not maximized, so that it can risk reducing future productivity levels [1].

In 2020, the distribution rate of the three growth problems in toddlers in Indonesia will already be in the acute chronic category. According to the World Health Organization (WHO), the maximum number of public health problems in a country should not exceed 20% to avoid problems that can be considered chronic [2]. Southeast Sulawesi is one of the provinces with under-five nutritional status problems that fall into the chronic-acute category, where the distribution rate of stunting is 30.2%, underweight is 20.9%, and wasting is 6.6%. With the high distribution of nutritional status problems, research is needed to determine the factors that affect the nutritional status of toddlers in Southeast Sulawesi.

Nutritional status in toddlers is the state of the body as a result of food consumption and the use of nutritious substances, differentiated between undernutrition, good nutrition, and overnutrition [3]. There are several basic factors that influence the nutritional status of toddlers, namely food, service, and practice factors. Food factors include the basic nutritional needs of pregnant women. Then the service factor includes services for mothers and babies as an action for health checks. Then the nutritional status of toddlers is also influenced by practical factors but indirectly. Based on the existing factors, these factors cannot be measured empirically, so indicators are needed that can measure the influence of these factors using methods that can handle multidimensional problems.

Based on the above problems, a method is needed that can measure and explain the relationship between factors and indicators that affect the nutritional status of toddlers. Structural Equation Modeling (SEM) is a suitable method to handle existing problems. This is because SEM is a statistical method that can determine the pattern of relationships between construct variables or latent variables and their indicators [4]. The SEM method has several conditions that must be met, namely, multivariate normal distributed data, observations must be independent of each other, indicators must be reflective, and the number of sample units used must be large and free from multicollinearity. If there are assumptions that are not met, the statistical model obtained will be less good [5]. However, in reality, real data collection in the field often shows abnormal data patterns due to uneven data collection that causes outliers, and the number of samples obtained in the field does not meet the requirements. So that a method is needed that can overcome the limitations of these assumptions, a variant-based SEM method called Partial Least Square Structural Equation Modeling (SEM-PLS) was developed by Herman Wold in 1966 [6]. The use of the SEM-PLS method is more flexible and powerful than ordinary SEM because it can be done on a small amount of data and free from multivariate normal assumptions.

In SEM-PLS, the sample taken is assumed to come from a homogeneous population. However, in reality, the population is not always homogeneous, so it can lead to invalid conclusions. In this study, to overcome these problems, the Response Based Unit Segmentation in Partial Least Square (REBUS-PLS) and Finite Mixture Partial Least Square (FIMIX-PLS) methods were used, which are methods to detect and analyze unobserved heterogeneity. REBUS-PLS and FIMIX-PLS analyses are performed by segmenting the observation units and estimating the parameters of each local model on the segments formed.

This study will model the factors that influence the nutritional status of children under five in Southeast Sulawesi Province using the SEM-PLS approach, then use REBUS-PLS and FIMIX-PLS to group regions in Southeast Sulawesi Province based on the nutritional status of children under five in 2020.

In assessing their influence, all factors affecting health status cannot be measured directly because these factors are variables that cannot be observed directly (latent variables) and are built from several indicator variables. Structural Equation Modeling (SEM) is used to represent the relationship between these latent variables. This SEM model has often been used to examine the relationship between latent variables [5], [6], [7]. To find the appropriate segments, we combined the SEM-PLS method with the REBUS and FIMIX methods [8,9]. This procedure is performed by segmenting the observation units and estimating the parameters of each local model on the formed segments. For REBUS-PLS, observation units are grouped based on similar behavior or similar performance [7]. While the grouping of observation units in FIMIX-PLS are grouped based on the strata.

SEM is a multivariate technique that combines aspects of factor analysis and multiple regression that allows research to be conducted simultaneously to examine the relationship between latent variables and latent variables with measured variables. SEM can test the structural equation model, which is the relationship between endogenous latent variables and exogenous latent variables. In addition, it can be used to test the measurement model, namely the relationship between indicator variables and latent variables, which shows the magnitude of the correlation between the indicator and the latent variable it describes [6]. SEM is not used to design a theory but is more intended to examine and justify a hypothesis model based on the justification of the theory described in the form of a path diagram [7]. The SEM model has three main components, namely variables, models, and errors.

There are two types of variables in the SEM method, namely latent variables and manifest variables. Latent variables are variables that cannot be measured, while manifest variables are variables that can be measured empirically. Latent variables consist of two types of variables, namely exogenous latent variables (ξ) and endogenous latent variables (η) [10]. Exogenous latent variables are independent variables in an equation, and endogenous latent variables are dependent variables in an equation.

In the SEM method, there are two types of models, namely structural models and measurement models. The structural model is a model that describes the relationship between latent variables. The measurement model is a model that describes the relationship between latent variables and manifest variables. The measurement model is carried out by factor analysis, i.e., each latent variable is modeled as a general factor of its measurement. The SEM method has two types of errors, namely structural error and measurement error.

## Structural equation modeling partial least square (SEM-PLS)

SEM-PLS is an alternative SEM approach that shifts from covariance-based to variance-based. The SEM-PLS design is intended to overcome the limitations of the SEM method when data experience problems such as measuring data with a certain scale, small sample size, missing values, abnormal data, and multicollinearity. In addition, SEM-PLS can be used on any type of data scale (nominal, ordinal, interval, or ratio) and has more flexible assumption requirements [8]. The measurement model consists of two models, namely the reflexive model and the formative model. The reflexive model is the relationship between manifest variables and latent variables, where if there is a change in the latent variable, it will result in a change in the manifest variable, but not vice versa. The reflexive model, also known as the principal factor model, has the following equation:

(1) $$x_{px1}=\Lambda_{pxn}\xi_{nx1}+\delta_{px1}$$

(2) $$y_{qx1}=\Lambda_{qxm}\eta_{mx1}+{ɛ}_{qx1}$$

The formative model is the opposite of the reflexive model, where if there is a change in the indicator variable, there will be a change in the latent variable. The formative model equation is as follows:

(3) $$\xi_{j}=\sum_{h=1}^{J}\lambda_{jh}x_{jh}+\delta_{j}$$

(4) $$\eta_{i}=\sum_{j=1}^{I}\lambda_{ji}y_{ji}+\varepsilon_{i}$$

The structural model is a model that describes the relationships that exist between latent variables based on substantive theory. Inner models are usually referred to as inner relations. The inner model equation is as follows:

(5) $$\eta_{mx1}=\beta_{mxm}\eta_{mx1}+\Gamma_{mxn}\xi_{nx1}+\zeta_{mx1}$$

Parameter estimates are obtained through a three-stage iteration process consisting of a weight estimate, a path estimate, means, and location parameters. The first stage of the weight estimate is used to create a score of latent variables [4]. Weight estimates for parameters in the reflexive model for exogenous latent variables are obtained by the least squares method by minimizing the sum of squared errors $\delta_{jh}$, so that the weights for the exogenous reflexive model are obtained as follows:

(6) $${\hat{\lambda }}_{h}=\frac{\hat{\text{cov}}\left(x_{h},\xi \right)}{\hat{\text{var}}\left(\xi^{2}\right)}$$

Meanwhile, the weight of the endogenous reflexive model is as follows:

(7) $${\hat{\lambda }}_{h}=\frac{\hat{\text{cov}}\left(y_{h},\eta \right)}{\hat{\text{var}}\left(\eta^{2}\right)}$$

The parameter estimation of the formative model is as follows:

(8) $${\hat{\lambda }}_{h}={\left[\text{var}\left(x_{h}\right)\right]}^{-1}\text{cov}\left(x_{h},\xi \right)$$

Path estimation is used to obtain parameter coefficient values for each latent variable. Measurement model estimation yj from standardized latent variables (*ξj* - *mj*) with mean 0 and standard deviation = 1, estimated by a linear combination of the centre of manifest variables while structural model estimation is obtained from standardized latent variables (*ξj* - *mj*). The inner model weights *e_ji_* are selected through three schemes, the path schemes, centroid scheme and factor scheme.

The estimated means and location parameters are the regression constants of the indicators and other variables, with the following equation:

(9) $$\mu_{j}=\sum_{h=1}^{H}{\tilde{\lambda }}_{jh}{\bar{x}}_{j}$$

(10) $${\hat{\gamma }}_{j0}=\xi_{j}-\sum_{i=1}^{I}{\hat{\gamma }}_{ji}{\hat{\xi }}_{i}$$

## Bootstrap

The bootstrap method is a non-parametric estimation method that can estimate the parameters of a distribution, the variance of the sample median, and the error rate. In bootstrapping, the same steps are required repeatedly to estimate the shape of the sampling distribution. Sampling using the bootstrap method is carried out by returning the data sample (resampling with replacement). PLS with small samples requires resampling with the bootstrap standard error method to obtain significance values and estimates of the measurement model and structural model that converge by finding estimates of standard error [9].

### Hypothesis testing

The hypothesis tested is the hypothesis in the outer model (λ) and the hypothesis in the inner model (*β* and γ) based on the parameter estimation results so that it is known whether the variables have a significant effect or not. The hypothesis on the outer model (λ) is as follows.

H_0_ : $\lambda_{h}=0$, *h=*1,2,…,*p* (The loading factor of the h^th^ indicator is not significant in measuring the latent variable)
H_1_ : $\lambda_{h}\neq 0$, *h=*1,2,…,*p* (The loading factor of the h^th^ indicator is significant in measuring the latent variable.)

### Test statistics

(11) $$T=\frac{{\hat{\lambda }}_{h}}{se\left({\hat{\lambda }}_{h}\right)}$$

where ${\hat{\lambda }}_{h}$ is the estimated value of $\lambda_{h}$ and $se({\hat{\lambda }}_{h})$ is the standard error for ${\hat{\lambda }}_{h}$. The rejection region used is H0 rejected if $|T|>t_{\alpha \;/\;2,df}$ or p-value < α.

The hypothesis in the inner model based on parameters β and γ is as follows:

***Beta* Parameter (** β **)**

H_0_ : $\beta_{i}=0$ (The i^th^ endogenous latent variable is not significant in measuring other endogenous latent variables.)
H_1_ : $\beta_{i}\neq 0$ (The i^th^ endogenous latent variable is significant in measuring other endogenous latent variables.)

***Gamma* Parameter (** γ **)**

H_0_ : $\gamma_{i}=0$ (The i^th^ exogenous latent variable is not significant in measuring the endogenous latent variable.)
H_1_ : $\gamma_{i}\neq 0$ (The i^th^ exogenous latent variable is significant in measuring the endogenous latent variable.)

### Test statistics

(12) $$T=\frac{{\hat{\beta }}_{i}}{se\left({\hat{\beta }}_{i}\right)}\;\text{or}\;T=\frac{{\hat{\gamma }}_{i}}{se\left({\hat{\gamma }}_{i}\right)}$$

H0 rejected if $|T|>t_{\alpha \;/\;2,df}$ or p-value < α.

## SEM-PLS model evaluation

The model evaluation stage in SEM-PLS includes the evaluation of the measurement model (outer model) and the structural model (inner model). The evaluation of the measurement model is based on the following values:

1. Convergent validity is seen based on item reliability and construct reliability. Based on item reliability, a correlation can be said to meet convergent validity if it has a loading factor value greater than 0.5 to 0.6 and the t-test value obtained from the bootstrapping process is greater than the t-table at a certain α, as in this study using α = 10% [8]. Meanwhile, based on construct reliability, it is expected that the Cronbach alpha value is > 0.7 for all constructs. The Cronbach alpha value is obtained from
  (13) $$ac=\frac{\sum_{\underset{h\neq h^{\prime }}{h=1}}^{p}cor\left(x_{h},x_{h^{\prime }}\right)}{\text{var}\left(\sum_{h=1}^{p}x_{h}\right)}x\frac{p}{p-1}$$

where $\text{var}\left(\sum_{h=1}^{p}x_{h}\right)=p+\sum_{\underset{h\neq h^{\prime }}{h=1}}^{p}cor(x_{h},x_{h^{\prime }})$

2. Discriminant The validity of the reflexive measurement model can be calculated based on the cross-loading value of the manifest variable on each latent variable. In addition, discriminant validity can also be calculated using the square root value of average variance extracted (AVE), which can be said to be good if the AVE is greater than 0.5.
  (14) $$AVE=\frac{\sum_{i=1}^{n}\lambda_{i}^{2}}{\sum_{i=1}^{n}\lambda_{i}^{2}+\sum_{i=1}^{n}\text{var}\left(\varepsilon_{\left(i\right)}\right)}$$
3. Composite reliability: latent variables can be said to have good reliability if the composite reliability value is greater than 0.6.
  (15) $$pc=\frac{{\left(\sum_{i=1}^{n}\lambda_{i}\right)}^{2}}{{\left(\sum_{i=1}^{n}\lambda_{i}\right)}^{2}+\sum_{i=1}^{n}\text{var}\left(\varepsilon_{\left(i\right)}\right)}$$
4. Variance Inflation Factor (VIF): if the VIF value is above 10, it indicates that there is multicollinearity.
  (16) $$VIF_{j}=\frac{1}{1-R_{j}^{2}}$$

The structural model was evaluated using the following statistical measures:

1. R-squared (R^2^): an R^2^ value of 0.75 can be categorized as strong or indicates that the model is well formed. Meanwhile, an R^2^ value of 0.5 is categorized as moderate (medium), and an R^2^ value of 0.25 means the model is categorized as weak [10].
  (17) $$R^{2}=\sum_{h=1}^{H}{\hat{\beta }}_{jh}cor\left(x_{jh},y_{j}\right)$$
2. Q-square Predictive relevance is used to validate the predictive ability of the model. If this value is greater than 0, it indicates that the exogenous latent variable is suitable as an explanatory variable that can predict the endogenous variable [8].
  (18) $$Q^{2}=1-\left(1-R_{1}^{2}\right)\left(1-R_{2}^{2}\right)...\left(1-R_{i}^{2}\right)$$

The evaluation of the overall measurement and structural models can be seen based on the goodness of fit index (GoF index) value as follows:

(19) $$GoF=\sqrt{\bar{communality}x{\bar{R}}^{2}}$$

The model is said to be able to explain empirical data well if the GoF value is greater than 0.36 [11].

## Agglomerative hierarchical cluster analysis

The agglomerative hierarchical cluster method is a clustering technique that starts with individual objects. These objects are then grouped based on similarity or dissimilarity until each object forms a cluster with similar characteristics in each cluster. The results of the method can be depicted in a two-dimensional diagram called a dendrogram. A dendrogram is a diagram that illustrates the stages of the hierarchical clustering process. The vertical axis of the dendrogram represents the homogeneity value or distance between groups, and the horizontal axis represents the grouped objects. The grouping is based on the distance between objects, which can be divided into four methods: single linkage, complete linkage, and average linkage [12].

## Metode ward's

Ward's method is a hierarchical clustering method that is based on minimizing information lost due to merging two groups. Ward's method, in other words, minimizes the increase in the sum square of error (SSE) criterion. The two groups that have the minimum increase in SSE will be grouped. When all groups combine into one group of N objects, calculating the distance between the two groups using Ward's method is as follows [12].

(20) $$SSE=\sum_{j=1}^{N}{\left(x_{j}-\bar{x}\right)}^{\prime }\left(x_{j}-\bar{x}\right)$$

## Response based unit segmentation in partial least square (REBUS-PLS)

REBUS-PLS is an advanced method of SEM-PLS that can group or segment observation units as well as estimate the parameters of each local model in the segments formed. The unit of observation is expressed based on similarities in behavior or performance. SEM-PLS assumes that the samples taken come from a homogeneous population. In reality, statistical data is not always homogeneous, which can lead to biased analysis results that result in invalid conclusions. REBUS-PLS is a method that can detect heterogeneity, where heterogeneity can be detected by grouping observation units. Grouping of observation units is based on a measure of closeness or distance of an index called the closeness size index (CM index), which is a goodness of fit index (GoF index) structure calculated from the residual commonality model described in the following equation.

(21) $$CM_{ig}=\sqrt{\frac{\sum_{j=1}^{J}\sum_{q=1}^{Pj}\left[e_{iqjg}^{2}/com\left({\hat{\xi }}_{jg},x_{qj}\right)\right]}{\frac{\sum_{i=1}^{N}\sum_{j=1}^{J}\sum_{q=1}^{Pj}\left[e_{iqjg}^{2}/com\left({\hat{\xi }}_{jg},x_{qj}\right)\right]}{\left(n_{g}-m_{g}-1\right)}}\times \frac{\sum_{j*=1}^{J*}\left[f_{ij*g}^{2}/R^{2}\left({\hat{\xi }}_{j*},{\hat{\xi }}_{j}\right)\right]}{\frac{\sum_{i=1}^{N}\sum_{j*=1}^{J*}\left[f_{ij*g}^{2}/R^{2}\left({\hat{\xi }}_{j*},{\hat{\xi }}_{j}\right)\right]}{\left(n_{g}-m_{g}-1\right)}}}$$

where $e_{iqjg}^{2}$ is the model residual size for the i^th^ unit in the g^th^ latent class, which refers to the q^th^ indicator variable in the j^th^ block, while $com({\hat{\xi }}_{jg}x_{qj})$ explains the communal index of the q^th^ variable from the j*^th^ block in the g^th^ latent class. In addition, $f_{ij*g}^{2}$ shows the residual of the structural model for the i^th^ unit in the g^th^ latent class, which refers to the j*^th^ endogenous block, *R*^2^ is the coefficient of determination from the model equation, and *n_g_* shows the number of units from the g^th^ latent class and *m_g_* shows the number dimensions [13].

To obtain a better local model than the global model, the selected closeness measure is determined according to the structure of the Goodness of Fit (GoF) index. The GoF index, according to Trinchera (2007), is defined as follows:

(22) $$GoF=\sqrt{\frac{\sum_{j=1}^{J}\sum_{q=1}^{Pj}cor^{2}\left(x_{qj},{\hat{\xi }}_{j}\right)}{\sum_{j=1}^{J}p_{j}}\times \frac{\sum_{j*=1}^{J*}R^{2}\left({\hat{\xi }}_{j*},{\hat{\xi }}_{j}\right)}{J*}}$$

where J is the number of latent variables in the model, and J* is the number of endogenous latent variables in the model. $cor(x_{qj},{\hat{\xi }}_{j})$ is the correlation between the q^th^ manifest variable of the j^th^ block and the corresponding score of the latent variable.

Clustering in REBUS-PLS aims to overcome unobserved heterogeneity by determining groups or clusters of observation units with similar behaviour and performance. In addition, because CM is defined based on the structure of the GoF index, the local model formed will show greater GoF and R^2^ values. The following are the steps in the REBUS-PLS method.

1. Calculate the structural and communality residuals from the global model to obtain initial groups or classes. The initial groups or classes were obtained based on the results of hierarchical cluster analysis of the remaining structural communalities in the form of a two-dimensional diagram, or dendrogram.
2. Calculate the local model of each class or segment formed using SEM-PLS analysis.
3. Recalculate the communality and structural residuals of each observation unit in each class formed. Then the CM value of each unit of each local model can be obtained.
4. Insert the observation units into the class that shows a smaller CM value. If the composition of observation units in a class changes, the number of local model classes also changes and is re-estimated using SEM-PLS.

Iteration continues until there is no change in the composition (observations included in the group or class) of the class or until the stop rule is reached. According to Trinchera (2007), the stop rule is when the difference in class composition is less than 0.05%, and usually, the iteration will converge in less than 15 iterations.

## Finite mixture partial least square (FIMIX-PLS)

In SEM-PLS applications, uncovering unobserved heterogeneity is critical to the quality of result presentation and interpretation [14]. Becker [15] explained that SEM is usually used for research purposes to model relationships between latent and manifest variables. Most of its applications assume that the data comes from a homogeneous population. However, the assumption of homogeneity is unrealistic. Each individual has different perceptions and evaluations of latent variables. The analysis applied if it does not pay attention to these segmentation differences will result in biased analysis, and the conclusions drawn will be invalid, so a method that can detect heterogeneity problems in the SEM model is needed.

Hahn et al. (2002) [16] developed the FIMIX-PLS method. This approach combines a finite mixture procedure with an EM algorithm specifically related to ordinary least squares (OLS) based on PLS predictions. Sarstedt (2008) [17] reviewed segmentation techniques for PLS path modelling and stated that FIMIX-PLS can be used as a comprehensive approach to determine heterogeneity in PLS path modeling. The statistical criterion used to indicate the best number of segments in FIMIX-PLS is known as entropy (EN). EN is a criterion used to analyze the class specification results of FIMIX-PLS, whose value is between 0 and 1. The higher the EN value, that is, the closer to 1, the better the quality of the separator and the model can be interpreted [16]. The assumption in FIMIX-PLS is that if the observation units have been separated according to their strata, then the case of heteroginity will not occur in the structural model.

The equation formed based on the relationship contained in the inner model is as follows:

(23) $$B\eta_{i}+\Gamma \xi_{i}=\zeta_{i}$$

FIMIX-PLS estimation is based on the assumption that heterogeneity occurs in the structural model, assuming *ηi* is finite mixture distributed with a multivariate normal density function $f_{i|k}\left(.\right)$.

(24) $$\eta_{i}∼\sum_{k=1}^{K}\rho_{k}f_{i|k}\left(\eta_{i}|\xi_{i},B_{k},\Gamma_{k},\Psi_{k}\right)$$

with

$\eta_{i}$: endogenous variable vector in the inner model (i = 1, 2, …, I)
$\rho_{k}$: the mixing proportion of latent class k, where $\rho_{k}>0$ and $\sum_{k=1}^{K}\rho_{k}=1$
$f_{i|k}\left(.\right)$: odds for case i given class k and parameter (.)
$B_{k}$: path coefficient matrix in the inner model for latent class k that shows the relationship between endogenous latent variables of size M x M
$\Gamma_{k}$: path coefficient matrix in the inner model for latent class k that shows the relationship between exogenous and endogenous latent variables of size M x J
$\Psi_{k}:\;$ M x M matrix for latent class k containing regression variance
*I*: total number of cases or observations
*i*: case or observation i (i = 1, 2, 3, …, I)
K: total number of classes
*k:* k classes or segments with *k* = 1, 2, 3, …, K

Model estimation in FIMIX-PLS follows the likelihood principle. The likelihood function in FIMIX-PLS is maximized by the Expectation-Maximization (EM) algorithm. The EM algorithm is a combination of the expectation (E) step and the maximization (M) step. The E-step produces an expected log-likelihood function that is used for parameter estimation. The M-step calculates the parameters by maximizing the expected log-likelihood of the E-step [4].

The optimum number of segments in FIMIX-PLS cannot be known for several reasons, including that the mixture model is not asymptotically chi-square distributed and valid for likelihood ratio tests. Hahn et al. (2002) suggested repeating the FIMIX-PLS operation with consecutive numbers of latent classes K (e.g., 1–10). Criteria such as Akaike's Information Criterion (AIC), Bayesian Information Criterion (BIC), Consistent AIC (CAIC), and Normed Entropy Criterion (EN) are used to determine the number of data segmentations. The formulas used to calculate the AIC, CAIC, BIC, and EN criteria are as follows [16].

(25) $$AIC_{k}=-2\;ln\;L+c\;N_{k}$$

where c is a constant, and $N_{k}$ is the number of parameters.

(26) $$N_{k}=\left(K-1\right)+KR=KQ$$

R is the number of predictor variables in the inner model regression model.

(27) $$BIC_{k}=-2\;ln\;L+\ln IN_{k}$$

in this equation the value of c in Eq. (25) is lnI.

(28) $$CAIC_{k}=-2\;ln\;L+\left(\ln\left(I\right)+1\right)\;N_{k}$$

in the above equation the value of c in Eq. (25) is (ln(I)+1)

(29) $$EN_{k}=1-\frac{[\sum_{i}\sum_{k}-P_{ik}\ln\left(P_{ik}\right)}{I\;\text{ln}\left(K\right)}$$

with

EN: Normal Entrophy, relative size between 0-1
$P_{ik}$: probability of the i^th^ observation in the k^th^ class
k: class or segment with k = 1, 2, …, K
i: i^th^ observation with i = 1, 2, …, I

## Research methodology

**Fig. 1:**
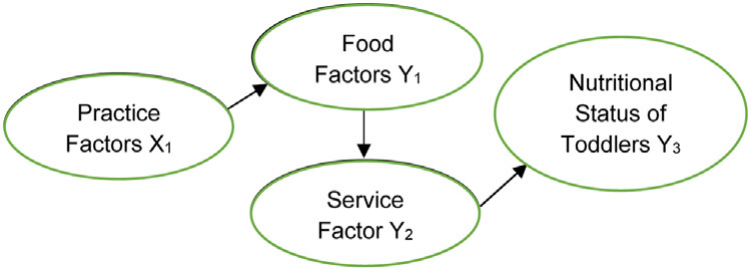
Conceptual framework for toddler nutrition status [18].

This study uses secondary data obtained from the Southeast Sulawesi Provincial Health Office 2020 on the nutritional status of children under five. The unit of observation used is the sub-district level in Southeast Sulawesi Province, which consists of 216 sub-districts. The research conceptual framework is presented in the following figure:

In the conceptual framework of Nutritional Status of Toddlers according to UNICEF in 2020 in Fig. 1, there are three basic factors that can affect the nutritional status of toddlers, namely food factors, service factors, and practice factors. This study uses three basic factors with indicators from each factor tailored to the variables in the research data. The nutritional status of toddlers in the study used was stunting, underweight, and wasting. Indicators of the food factor are pregnant women getting Fe1 tablets and Fe3 tablets. Indicators of the service factor are babies with low birth weight, pregnant women with chronic energy deficiency, anaemic pregnant women, and complete neonatal visits. Indicators from the practice factor of the number of posyandu will help mothers and babies do health checks to avoid malnutrition. Details are presented in the following table.

The analysis step carried out in this study consists of two stages, namely the modeling analysis stage using SEM-PLS to answer the first research objective and the clustering analysis stage using the REBUS-PLS method to answer the second research objective.

The first-stage analysis steps using the SEM-PLS method are as follows:

1. Describe the characteristics of data based on descriptive statistics.
2. Designing structural models and measurement models. The design of the structural model of the relationship between latent variables is based on the formulation of the problem or research hypothesis, while the design of the measurement model determines the nature of the indicators of each latent variable based on the operational definition of the variable.
3. Constructing a path diagram that explains the relationship pattern between latent variables and their indicators.
4. Evaluating the measurement model, followed by evaluating the structural model.
5. Estimating outer model and inner model parameters.
6. Hypothesis testing of measurement models and structural models using the bootstrap resampling method with hypothesis and t-test statistics.

After obtaining the model with the SEM-PLS method, segmentation is continued with the REBUS-PLS and FIMIX-PLS methods. The REBUS-PLS method analysis steps are as follows:

1. Conduct agglomerative hierarchical cluster analysis based on structural and communal residuals from the global model of the first-stage analysis results.
2. Selecting the number of segments or clusters based on the dendrogram formed.
3. Grouping each case into segments according to agglomerative hierarchical cluster analysis
4. Estimating the local model in each segment using SEM-PLS
5. Calculating the closeness measure index (CM index) for each case in each local model
6. Clustering each case or observation unit based on the smallest CM index value formed and recalculating the local model for each cluster until the cluster composition does not change.
7. Describe the classes obtained according to the differences between local models.
8. Comparing the analysis results on the global model and local model
9. Draw conclusions and make suggestions.

Next is to conduct clustering analysis with the FIMIX-PLS approach.

1. Estimating the path model based on the PLS algorithm.
2. The factor score value of a significant model is obtained from the latent variable factor score value in the inner model. The factor score value in the inner model is used for the FIMIX-PLS procedure, namely determining the number of groups.
3. Expost analysis and assessment of explanatory variables for segmentation.
4. Comparing the previous data segmentation with the predicted results of the path model.
5. Evaluating and interpreting the results of PLS path segmentation.
6. Draw conclusions and make suggestions.

## Results

Descriptive statistics will be used to determine the characteristics of each factor affecting the nutritional status of children under five in Southeast Sulawesi in 2020, with 216 sub-districts as observation units. The following are the characteristics of the factors that affect the nutritional status of children under five.

Table 2 shows that the average value of the three nutritional status problems of children under five years old is stunting, with the highest number of cases, followed by underweight and wasting. Stunting has an average number of cases of 68.866, wasting is 19.278 cases, and underweight is 49.042. This shows that the number of cases of under-five nutritional status problems in Southeast Sulawesi Province is still quite large. The sub-districts with the highest number of cases are the Mawasangka sub-district for stunting cases, the Binongko sub-district for wasting cases, and the Pasar Wajo sub-district. Then there are 29 sub-districts with 0 stunting cases, 33 sub-districts with 0 wasting cases, and 27 sub-districts with underweight cases.

**Table 1:** Operational definition of research variables.

Latent Variable		Indicator	Scale
Food Factors	Y_1,1_	Number of pregnant women receiving Fe1 tablets	Ratio
	Y_1,2_	Number of pregnant women receiving Fe3 tablets	Ratio
Service Factors	Y_2,1_	Number of babies with low birth weight (LBW)	Ratio
	Y_2,2_	Number of pregnant women with Chronic Energy Deficiency (CHD)	Ratio
	Y_2,3_	Number of pregnant women with anemia	Ratio
	Y_2,4_	Number of complete Neonate visits	Ratio
Nutritional Status of Toddlers	Y_3,1_	Number of stunted toddlers	Ratio
	Y_3,2_	Number of underweight toddlers	Ratio
	Y_3,3_	Number of children under five wasting	Ratio
Practice Factors	X_1,1_	Number of posyandu	Ratio

**Table 2:** Descriptive statistics based on research variable indicators.

Variable	Mean	Variance	Min	Max
Number of posyandu (X1.1)	9.231	82.228	0	54
Number of pregnant women receiving Fe1 tablets (Y1.1)	228.102	42779.89	9	1413
Number of pregnant women receiving Fe3 tablets (Y1.2)	195.052	38430.51	11	1437
Number of babies with low birth weight (LBW) (Y2.1)	5.870	53.071	0	43
Number of pregnant women with Chronic Energy Deficiency (CHD) (Y2.2)	37.079	1318.997	0	228
Number of pregnant women with anemia (Y2.3)	66.736	10628.17	0	679
Number of complete Neonate visits (Y2.4)	206.505	33546.12	0	1364
Number of stunted toddlers (Y3.1)	68.866	5935.470	0	434
Number of underweight toddlers (Y3.2)	19.278	523.219	0	137
Number of children under five wasting (Y3.3)	49.042	3398.307	0	366

The number of posyandu in Southeast Sulawesi Province as a manifest variable of the latent variable practice factor has an average of 9.231, so it can be said that the number of posyandu in each sub-district is quite low. There are 77 out of 216 sub-districts that do not have posyandu, and the sub-district with the highest number of posyandu is the Wangi-Wangi sub-district, with 54 existing posyandu.

## SEM-PLS analysis

**Fig. 2:**
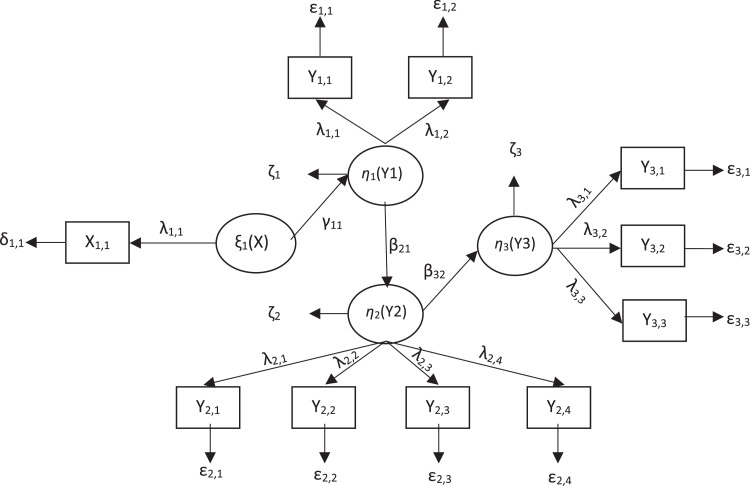
Path diagram of the research model.

PLS-SEM analysis begins by forming a path diagram by designing a measurement model and structural model first in accordance with the conceptual framework that has been built. In this study, the structural model consists of 1 exogenous latent variable, namely the practice factor (ξ1) or (X), and 3 endogenous latent variables, namely food factors (η1) or (Y1), service factors (η2) or (Y2), and nutritional status of toddlers (η3) or (Y3). The measurement model in this study uses a reflective measurement model where manifest variables are influenced by latent variables. Furthermore, the relationship between latent variables and their indicators and the relationship between exogenous and endogenous latent variables is described through a path diagram. The path diagram can be shown in Fig. 2 below.

Measurement model analysis consists of testing the validity and reliability of each indicator against its latent variable or measurement model evaluation. Validity and reliability tests aim to determine whether the indicators used are valid and reliable for measuring each latent variable. The validity assessment is based on the value of the loading factor, which shows the correlation of each indicator with its latent variable. Discriminant validity is determined by looking at the square root value of the average variance extracted (AVE). If the AVE root value is > 0.50, it can be said that the latent variable has good discriminant validity. Reliability testing is carried out to determine the reliability of latent variables as a measuring instrument. Latent variables are said to have high reliability as measuring instruments if they have a Cronbach's alpha value ≥ 0.60 and composite reliability ≥ 0.70. The measurement model estimation results are presented in the following table.

Table 3 shows that all indicators of each latent variable have a factor loading value above 0.5 with a T-statistic greater than t-table = 1.96 and a p-value <0.05, so the indicator is valid and significant. Furthermore, it also provides a composite reliability value above the cut-off value of 0.7, so it can be said that all latent variables are reliable. The practice factor (X) is formed by the indicator number of posyandu (X1.1). The food factor (Y1) is formed by the indicators of the number of pregnant women getting Fe1 tablets (Y1.1) (0.992) and the number of pregnant women getting Fe3 tablets (Y1.2) (0.992). Service Factor (Y2) dominant indicators are the number of complete neonate visits (Y2.4) (0.884), the number of pregnant women with chronic energy deficiency (CHD) (Y2.2) (0.752), and the number of babies with low birth weight (LBW) (Y2.1) (0.727). The dominant nutritional status of toddlers (Y3) is an indicator of the number of underweight toddlers (Y3.3) (0.958), stunting toddlers (Y3.1) (0.919), and wasting toddlers (Y3.2) (0.913).

**Table 3:** Indicator validity and reliability test on latent variables.

Latent Variable	Indicator	*Loading Factor*	Standard Deviation (STDEV)	T Statistics (|O/STDEV|)	P-Values	$\sqrt{AVE}$	*Cronbach's Alpha*	*Composite Reliability*
Practice Factors (X)	Number of posyandu (X1.1)	1.000				1.000	1.000	1.000
Food Factors (Y1)	Number of pregnant women receiving Fe1 tablets (Y1.1)	0.992	0.003	322.510	0.00	0.992	0.983	0.992
	Number of pregnant women receiving Fe3 tablets (Y1.2)	0.992	0.003	346.818	0.00			
Service Factors (Y2)	Number of babies with low birth weight (LBW) (Y2.1)	0.727	0.056	12.887	0.00	0.744	0.730	0.829
	Number of pregnant women with Chronic Energy Deficiency (CHD) (Y2.2)	0.752	0.050	15.144	0.00			
	Number of pregnant women with anemia (Y2.3)	0.579	0.075	7.701	0.000			
	Number of complete Neonate visits (Y2.4)	0.884	0.016	55.658	0.000			
Nutritional Status of Toddlers (Y3)	Number of stunted toddlers (Y3.1)	0.919	0.017	54.291	0.000	0.930	0.992	0.951
	Number of underweight toddlers (Y3.2)	0.913	0.010	97.577	0.000			
	Number of children under five wasting (Y3.3)	0.958	0.019	48.952	0.000			

The results of the original estimation and the bootstrap estimation, B = 500, are presented in the following figure.

The results of testing the complete model can be seen from the R-Square value, which describes the goodness-of-fit of a model. The recommended R-square value is greater than zero. The R-square value is presented in Table 4 below:

Table 4 explains that the contribution or proportion of the variable Practice Factor (X) to the Food Factor (Y1) is 0.148, the variable Food Factor (Y1) to the Service Factor (Y2) is 0.686, and the Service Factor (Y2) to the Nutritional Status of Toddlers (Y3) is 0.078. The results of all R-Square values show that all R-Square values are greater than zero. This means that this research model has met the required Goodness of Fit. Based on the f2 value, it can be seen that the service factor latent variable has a small influence, the food factor latent variable has a large influence, and the practice factor latent variable has a moderate influence.

**Table 4:** The goodness of fit of the R-square model of toddler nutrition status.

Variable	*R^2^*	*f*^2^
Practice Factors (X) → Food Factors (Y1)	0.148	0.179
Food Factors (Y1) → Service Factors (Y2)	0.686	2.202
Service Factors (Y2) → Nutritional Status of Toddlers (Y3)	0.078	0.090

Source: Data processed.

The results of the calculation of the Q-square value from Table 4 are as follows:

$$Q^{2}=1-\left(1-0.148\right)\times \left(1-0.686\right)\times \left(1-0.078\right)=0.753$$

**Table 5:** Coefficient of structural model parameters.

	Original Sample (O)	Sample Mean (M)	Standard Deviation (STDEV)	T Statistics (|O/STDEV|)	P- Values
Practice Factors (X) → Food Factors (Y1)	0.390	0.381	0.095	4.104	0.000
Food Factors (Y1) → Service Factors (Y2)	0.829	0.833	0.024	34.855	0.000
Service Factors (Y2) → Nutritional Status of Toddlers (Y3)	0.287	0.290	0.070	4.106	0.000

It can be interpreted that the model can explain the nutritional status of toddlers (Y3) by 75.3%, and 24.7% is explained by other variables outside the model. The structural equation is presented below:

FoodFactors(Y1)=0.390PracticeFactors(X)

ServiceFactors(Y2)=0.829FoodFactors(Y1)

NutritionalStatusofToddlers(Y3)=0.287ServiceFactors(Y2)

**Fig. 3:**
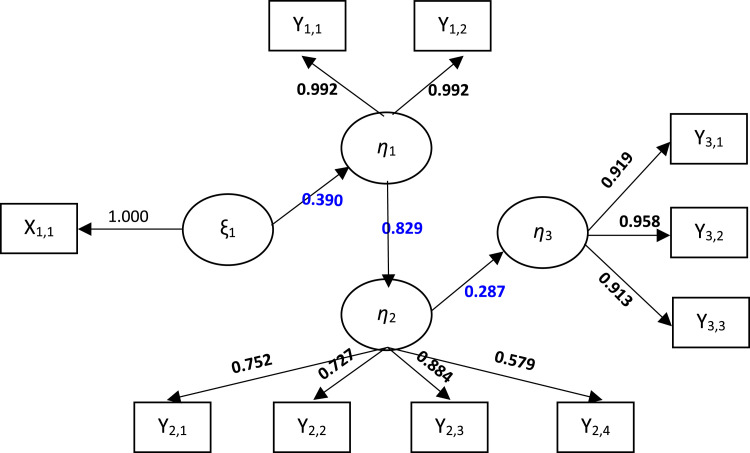
Path coefficient on the global model with SEM-PLS.

The path coefficient test in Fig. 3 and the above equation in detail are presented in the following table:

Based on Table 5 and the equation above, it can be interpreted as follows:

1. Practice factors have a significant and positive effect on food factors. This can be seen from the positive path coefficient of 0.390 with a T-statistics value of 4.104, which is greater than the t-table of 1.96 or p-value of 0.000, which is smaller than the significance level (α) determined at 0.05. Thus, the practice factor has a direct effect on the food factor of 0.390, which means that every increase in the practice factor will increase the food factor by 0.390.
2. Food factors have a significant and positive effect on service factors. This can be seen from the positive path coefficient of 0.829 with a T-statistics value of 34.855, which is greater than the t-table of 1.96, or the p-value of 0.000, which is smaller than the significance level (α) determined at 0.05. Thus, the food factor has a direct effect on the service factor of 0.829, which means that every increase in the food factor will increase the service factor by 0.829.
3. Service factors have a significant and positive effect on the nutritional status of children under the age of five. This can be seen from the positive path coefficient of 0.287 with a T-statistics value of 34.855, which is greater than the t-table = 1.96, or p-value = 0.000, less than the significance level (α) determined at 0.05. Thus, the service factor has a direct effect on the nutritional status of children under five by 0.287, which means that every increase in the service factor will increase the nutritional status of children under five by 0.287.

PLS-SEM analysis assumes that there is heterogeneity in the observation data used; to overcome this, the REBUS-PLS and FIMIX-PLS methods are used. The initial stage in the REBUS-PLS analysis is to obtain the initial class and calculate the Closeness Measure Index (CM Index) value for each class formed, while in FIMIX-PLS, the Normed Entropy Criterion (EN) is used to determine the number of data segmentations. Initial class initialization used Ward's method of cluster analysis.

The initial class is obtained based on the results of hierarchical cluster analysis using Ward's method in the form of a dendrogram.

Determining the best number of clusters formed is based on the following statistical criteria:

Table 6 shows that the best number of clusters for the REBUS-PLS method is 2 clusters. This can be seen from the GQI value; the GQI Inner model provides the highest value. While in the FIMIX-PLS method, the best number of clusters is 2 clusters, this can be seen from the EN value in 2 clusters giving the highest value, which is 0.738.

**Table 6:** Cluster determination criteria from the results of Ward's method.

Criteria	REBUS-PLS				Criteria	FIMIX-PLS			
	2	3	4	5		2	3	4	5
GQI	0.567	0.535	0.476	0.480	AIC	1328.9	1314.7	1287.4	1302.2
GQI *Outer Model*	0.853	0.863	0.817	0.823	BIC	1372.8	1382.2	1378.5	1417.0
GQI *Inner Model*	0.665	0.620	0.583	0.583	CAIC	1346.6	1402.2	1405.5	1451.0
GoF	0.481	0.481	0.481	0.481	EN	0.738	0.723	0.723	0.648

Grouping observations in each cluster were formed based on the Closeness Measure Index (CM Index) value for the REBUS-PLS method and the EN value for FIMIX-PLS. Grouping is done by comparing the CM index values between segments; the segment with the smallest CM index value indicates that the observation is included in the segment. EN is a criterion used to analyze the results of class specifications from FIMIX-PLS, whose value is between 0 and 1. A higher EN value indicates that the observation is included in the segment (see Appendix 1).

Based on the grouping results using the CM value, in segment 1, there are 75 sub-districts, and in segment 2, there are 141 sub-districts. The characteristics for each segment based on descriptive statistics are presented as follows:

After cluster analysis, observations are grouped based on the probabilities of segment membership values. Grouping is done by comparing the probabilities of segment membership values between segments; the segment with the smallest probability of segment membership value indicates that the observation is included in the segment. The following is a mapping of the results of the clustering of observation units based on the CM index value and the EN index.

After grouping observations based on the CM index value, the REBUS-PLS results for 2 clusters form local model 1 (L1) and local model 2 (L2), which are compared with the results of SEM-PLS analysis as the global model (GM). The following is the path diagram for GM, L1, and L2.

Then the REBUS-PLS results will be compared with the PLS-SEM analysis results to see the comparison of the analysis results using PLS-SEM and REBUS-PLS. The following is a comparison of path diagrams for GM, L1, and L2.

Based on Table 8, it can be seen from the path coefficient value that each relationship between latent variables has a positive influence. When viewed based on the path coefficient value for the relationship between practice factors and food factors, local model 1 has a greater path coefficient value than the global model. The relationship between food factors and service factors Local model 2 has a greater path coefficient value than the global model, and for the relationship between service factors and the nutritional status of toddlers, local model 2 has a greater path coefficient value than the global model. This shows that the results of the analysis using homogeneous data have greater results than heterogeneous data. Next, a comparison of loading factors between the global model, local model 1, and local model 2 is carried out.

**Table 7:** Descriptive statistics of indicators in segment 1 and segment 2: REBUS and FIMIX methods.

Indicator	REBUS-PLS				FIMIX-PLS			
	Mean		Variance		Mean		Variance	
	Segment 1	Segment 2	Segment 1	Segment 2	Segment 1	Segment 2	Segment 1	Segment 2
Number of posyandu (X1.1)	8.160	9.801	99.681	72.017	10.761	8.818	138.345	66.243
Number of pregnant women receiving Fe1 tablets (Y1.1)	359.387	158.270	73446.98	12423.43	488.087	157.753	77956.5	10023.213
Number of pregnant women receiving Fe3 tablets (Y1.2)	321.533	127.773	69228.84	9012.899	436.174	129.806	78824.5	7511.689
Number of babies with low birth weight (LBW) (Y2.1)	8.533	4.454	84.062	30.815	11.239	4.418	133.795	21.326
Number of pregnant women with Chronic Energy Deficiency (CHD) (Y2.2)	56.280	26.865	1938.148	742.443	77.935	26.024	2540.06	414.692
Number of pregnant women with anemia (Y2.3)	92.120	53.234	18834.78	5737.810	119.348	52.500	26008.7	5514.696
Number of complete Neonate visits (Y2.4)	346.920	131.816	49951.65	8754.037	419.935	148.753	73988.9	6941.722
Number of stunted toddlers (Y3.1)	48.080	55.660	3845.194	6695.347	122.152	54.447	14050.3	2763.605
Number of underweight toddlers (Y3.2)	11.973	23.163	2794.480	3593.288	101.870	34.747	8749.73	990.739
Number of children under five wasting (Y3.3)	36.600	79.922	52.863	651.498	38.022	14.206	1155.46	231.375

**Table 8:** Path coefficients for the global model, segment 1, and segment 2.

Variable	PLS	REBUS		FIMIX	
	GM (N=216)	Seg-1 (N=75)	Seg-2 (N=141)	Seg-1 (N=46)	Seg-2 (N=170)
Practice Factors → Food Factors	0.390	0.540	0.504	0.327	0.469
Food Factors → Service Factors	0.829	0.797	0.843	0.883	0.810
Service Factors → Nutritional Status of Toddlers	0.287	0.404	0.701	0.521	0.182

Table 9 shows that each indicator of both the global model and local model (segment) has a loading factor value > 0.5, so the indicators for the global model and local model are valid. Based on the comparison between the loading factor values of the global model and the local model, the loading factor value in the local model is greater than the global model, so it is concluded that the loading factor value using homogeneous data has greater results than heterogeneous data. Then the following is a comparison of R^2^ and Q^2^ values between the global model and the local model.

**Table 9:** Comparison of loading factors.

Latent Variable	Indicators	*PLS*	*REBUS*		*FIMIX*	
		GM (N=216)	Seg-1 (N=75)	Seg-2 (N=141)	Seg-1 (N=46)	Seg-2 (N=170)
Practice Factors	Number of posyandu (X1.1)	1.000	1.000	1.000	1.000	1.000
Food Factors	Number of pregnant women receiving Fe1 tablets (Y1.1)	0.992	0.991	0.985	0.987	0.984
	Number of pregnant women receiving Fe3 tablets (Y1.2)	0.992	0.992	0.983	0.989	0.981
Service Factors	Number of babies with low birth weight (LBW) (Y2.1)	0.727	0.692	0.722	0.682	0.594
	Number of pregnant women with Chronic Energy Deficiency (CHD) (Y2.2)	0.752	0.633	0.758	0.663	0.755
	Number of pregnant women with anemia (Y2.3)	0.579	0.590	0.543	0.670	0.680
	Number of complete Neonate visits (Y2.4)	0.884	0.894	0.786	0.936	0.820
Nutritional Status of Toddlers	Number of stunted toddlers (Y3.1)	0.919	0.891	0.921	0.931	0.904
	Number of underweight toddlers (Y3.2)	0.913	0.940	0.911	0.962	0.953
	Number of children under five wasting (Y3.3)	0.958	0.891	0.969	0.932	0.773

**Fig. 4:**
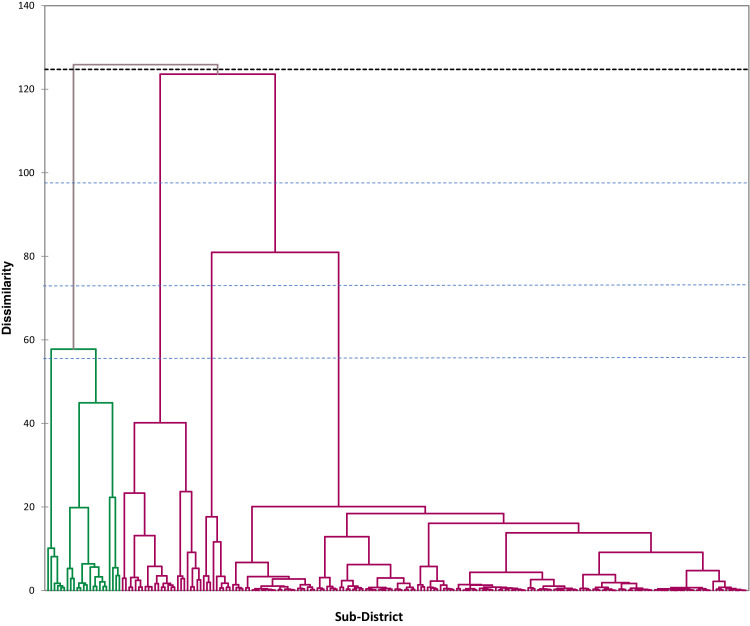
Dendrogram of cluster analysis results.

**Fig. 5:**
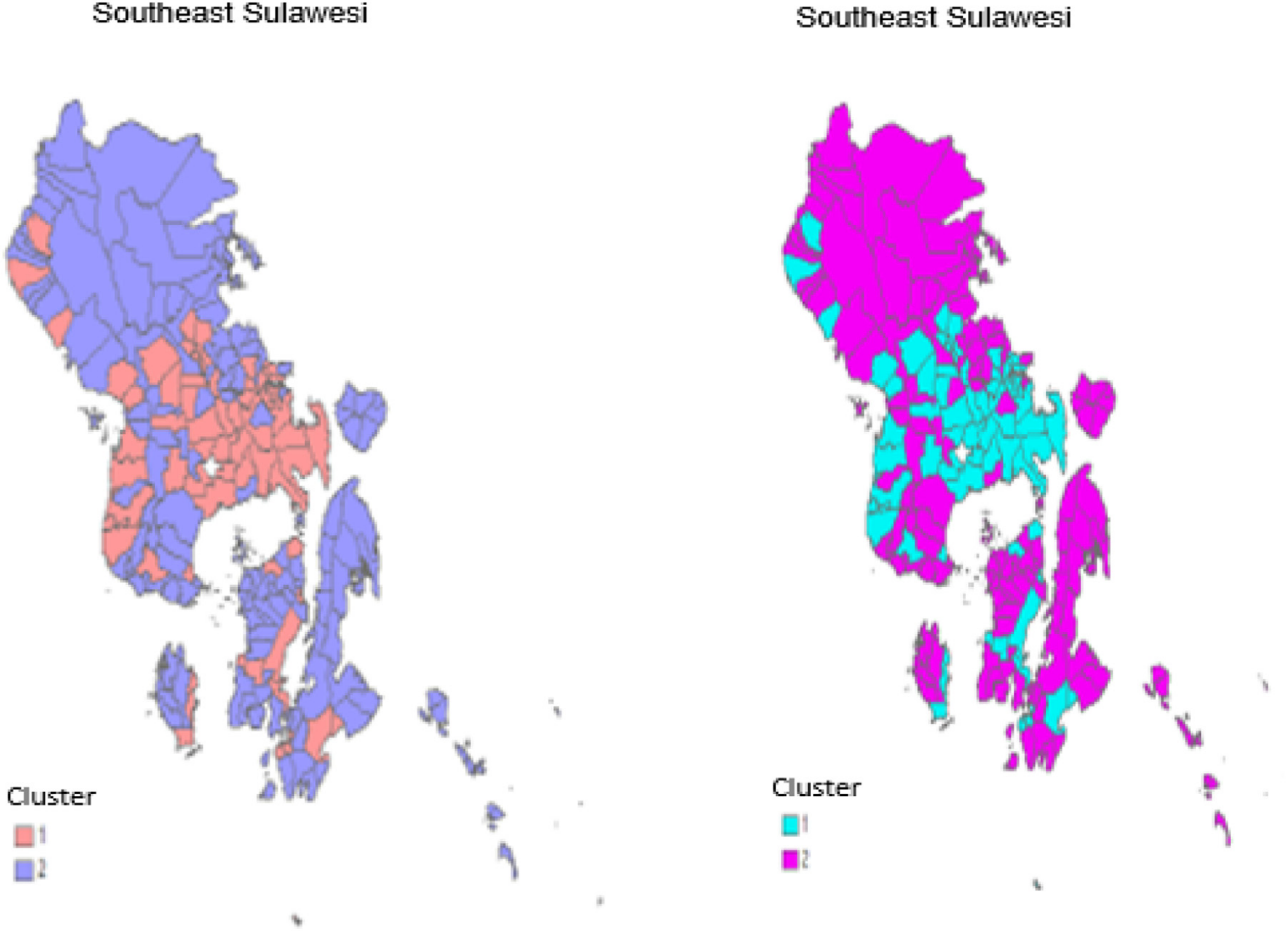
Mapping of cluster analysis results based on the CM index in Southeast Sulawesi.

**Fig. 6:**
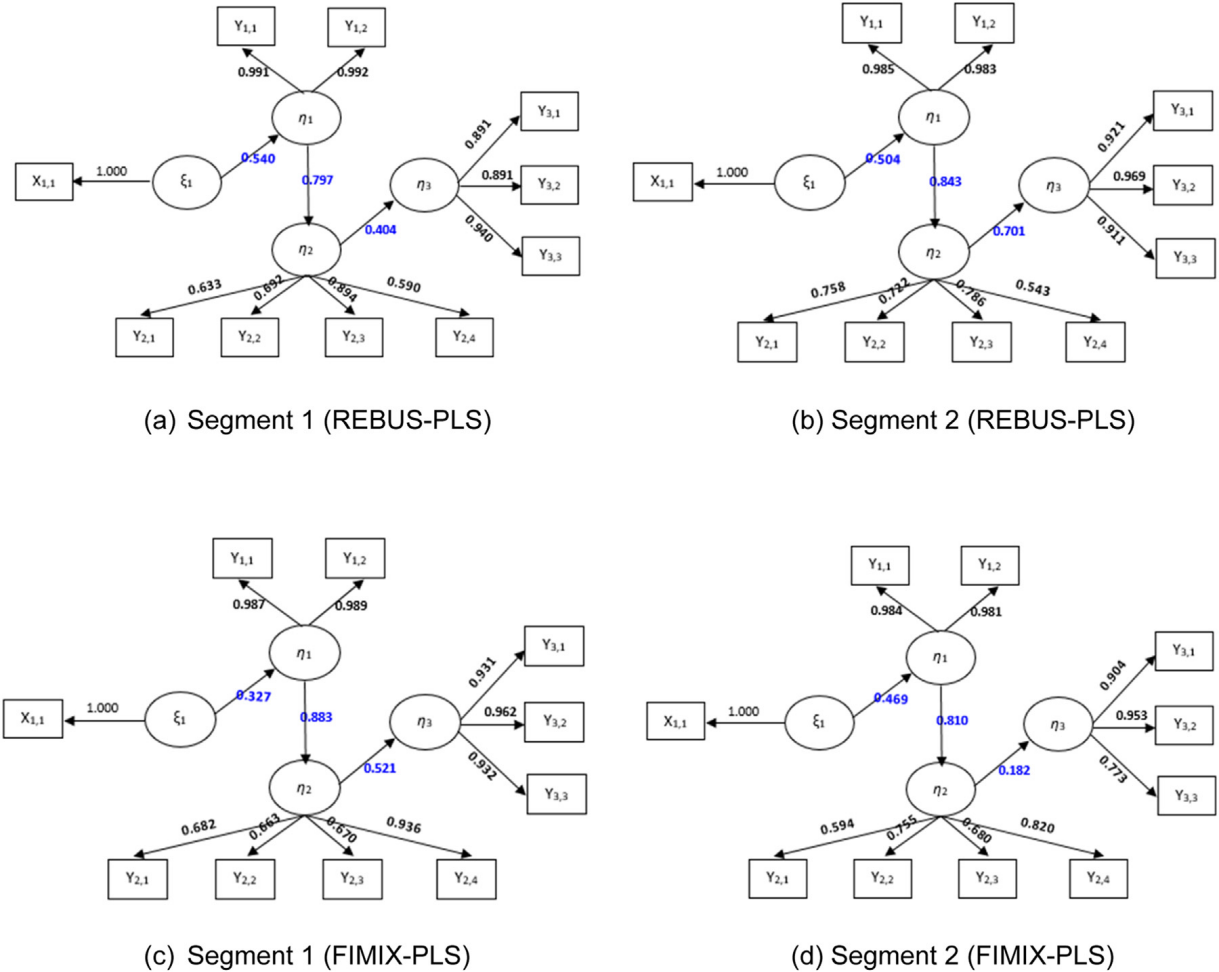
Path diagram of segmentation 1 and 2 of the REBUS-PLS and FIMIX-PLS methods.

Table 10 shows that the Q-square values in the REBUS and FIMIX method segments provide higher values than the PLS method, except for Segment 2 in the FIMIX method. Segment 1 in the REBUS and FIMIX methods has a larger R2 value than in the global model; this is in the food factor model that affects service factors. However, because the comparison between the R2 values for the local model and the global model is not much different, it does not mean that if the analysis is carried out using the original data without being grouped, it will produce poor results. However, analysis using data that has been grouped will produce greater results than without grouping, even though the difference is not so significant (Fig. 4, Fig. 5, Fig. 6, Tables 1, 7).

**Table 10:** Comparison of R-square and Q-square values.

Latent Variable	PLS	REBUS		FIMIX	
	GM (N=216)	Seg-1 (N=75)	Seg-2 (N=141)	Seg-1 (N=46)	Seg-2 (N=170)
Practice Factors (X) → Food Factors (Y1)	0.148	0.254	0.292	0.107	0.220
Food Factors (Y1) → Service Factors (Y2)	0.686	0.711	0.634	0.780	0.656
Service Factors (Y2) → Nutritional Status of Toddlers (Y3)	0.078	0.491	0.163	0.272	0.033
*Q-square*	0.753	0.890	0.783	0.857	0.740

## Conclusion

Based on the results of the analysis and discussion that have been carried out, it can be concluded as follows:

1. The results of the SEM-PLS analysis with the measurement model show that the Posyandu indicator is a valid, reliable, and significant indicator for describing the practice factor. Pregnant women receiving Fe1 tablets and pregnant women receiving Fe3 tablets are valid and significant indicators in describing food factors. Then, infants with low birth weight (LWB), pregnant women with chronic energy deficiency (CED), anemic pregnant women, and neonate visits are valid and significant indicators in describing the service factor. Stunting, wasting, and underweight are valid and significant indicators in describing the nutritional status of children under five. Then, the results of the analysis with the structural model show that the practice factor significantly affects the food factor with a path coefficient value of 0.390, the food factor significantly affects the service factor with a path coefficient of 0.829, and the service factor significantly affects the nutritional status of children under five with a path coefficient value of 0.287.
2. REBUS-PLS and FIMIX-PLS produce two segments, which is the number of best segments formed. REBUS-PLS: segment 1 consists of 75 sub-districts, and segment 2 consists of 141 sub-districts. In FIMIX-PLS, segment 1 consists of 46 sub-districts, and segment 2 consists of 170 sub-districts. In both analyzes using REBUS-PLS and FIMIX-PLS, the local model has an R2 value that is greater than the global model, even though there is a value in one local model that is smaller than the global model. For this variable, the other local model has a greater value compared to the global model. The REBUS-PLS method provides the best performance in dealing with heterogeneity compared to FIMIX-PLS and PLS.
3. The food factor has a dominant influence on the service factor; therefore, the indicator number of pregnant women receiving Fe1 tablets and Fe3 tablets is the indicator that has the greatest contribution in describing the service factor and then to the nutritional status of children under five, so that the indicator should be given more attention to reducing stunting, wasting, and underweight in Southeast Sulawesi. In addition, the different coefficient results in each model indicate different characteristics for each cluster formed and allow that there are other factors that can affect the occurrence of stunting, wasting, and underweight in each cluster.

## Ethics statements

The data used are secondary and primary data. Secondary data from the Southeast Sulawesi Provincial Health Office data in 2021 whose collection was carried out by data management officers. Health Office data was obtained by applying for permission from the head of the Southeast Sulawesi Provincial Health Office. Data collection is based on data presented at the aggregate district/city level such as data on the number of stunting cases, the number of wasting cases and the number of underweight cases, the percentage of toddlers of male sex, the percentage of toddlers aged 12-59 months, the percentage of BBLR, the percentage of anemic pregnant women, the percentage of 6-month-old babies exclusively breastfed, the percentage of toddlers with complete basic immunization, the percentage of toddlers receiving vitamin A, the percentage of postpartum mothers receiving vitamin A, the ratio of Active Posyandu, the percentage of visits by pregnant women K4, the percentage of toddlers suffering from pneumonia, the percentage of toddlers suffering from diarrhea, the percentage of households accessing proper sanitation facilities. Furthermore, primary data by conducting Focus Group Discussions (FGDs) confirming and validating research data with the Southeast Sulawesi Provincial Health Office [19].

## CRediT authorship contribution statement

**Bambang Widjanarko Otok:** Conceptualization, Visualization, Writing – original draft, Writing – review & editing. **Purhadi:** Conceptualization, Visualization, Writing – review & editing. **Riry Sriningsih:** Writing – review & editing. **Dalbergia Septi Dila:** Writing – original draft.

## Declaration of Competing Interest

The authors declare that they have no known competing financial interests or personal relationships that could have appeared to influence the work reported in this paper.

## Figures and Tables

**Table:** 

No	Sub-district	Seg1	Seg2	Seg	Seg1	Seg2	Seg	No	Sub-district	Seg1	Seg2	Seg	Seg1	Seg2	Seg

1	Abeli	0,221	1,079	1	0.219	0.781	2	109	Molawe	0,096	0,039	2	0.979	0.021	1
2	Abuki	0,352	0,095	2	0.982	0.018	1	110	Moramo	0,401	1,243	1	0.462	0.538	2
3	Aere	2,452	0,192	2	0.924	0.076	1	111	Morosi	0,120	0,155	1	0.969	0.031	1
4	Amonggedo	1,823	0,341	2	0.969	0.031	1	112	Motui	0,158	0,036	2	0.962	0.038	1
5	Andoolo	0,297	2,007	1	0	1	2	113	Mowewe	0,225	0,013	2	0.983	0.017	1
6	Andowia	0,219	0,052	2	0.974	0.026	1	114	Mowila	0,082	0,516	1	0.983	0.017	1
7	Angata	2,648	5,541	1	0	1	2	115	Murhum	0,898	1,434	1	0.912	0.088	1
8	Anggaberi	0,124	0,129	1	0.974	0.026	1	116	Nambo	0,879	0,451	2	0.869	0.131	1
9	Anggalomoare	0,176	0,056	2	0.949	0.051	1	117	Napabalano	0,113	0,522	1	0.972	0.028	1
10	Anggotoa	0,102	0,042	2	0.977	0.023	1	118	Napano Kusambi	0,272	0,089	2	0.973	0.027	1
11	Asera	0,362	0,184	2	0.977	0.023	1	119	Ngapa	0,112	2,880	1	0.904	0.096	1
12	Asinau	0,160	0,117	2	0.965	0.035	1	120	Oheo	0,185	0,117	2	0.977	0.023	1
13	Baito	0,128	0,280	1	0.969	0.031	1	121	Onembute	0,119	0,138	1	0.962	0.038	1
14	Barangka	0,174	0,066	2	0.98	0.02	1	122	Padangguni	0,330	0,108	2	0.957	0.043	1
15	Baruga	0,279	2,836	1	0.177	0.823	2	123	Pakue	0,115	0,083	2	0.961	0.039	1
16	Basala	0,224	0,012	2	0.985	0.015	1	124	Pakue Tengah	0,385	0,020	2	0.984	0.016	1
17	Batalaiworu	0,931	0,095	2	0.975	0.025	1	125	Pakue Utara	0,160	0,038	2	0.988	0.012	1
18	Batauga	4,681	1,268	2	0.747	0.253	1	126	Palangga	0,281	1,056	1	0.768	0.232	1
19	Batu Putih	0,162	0,199	1	0.97	0.03	1	127	Palangga Selatan	0,107	0,075	2	0.975	0.025	1
20	Batuatas	2,897	0,936	2	0.13	0.87	2	128	Palinggona	0,630	0,142	2	0.963	0.037	1
21	Batukara	0,138	0,094	2	0.973	0.027	1	129	Parigi	0,292	0,059	2	0.973	0.027	1
22	Batupoaro	0,175	0,638	1	0.279	0.721	2	130	Pasaw Wajo	4,973	6,448	1	0	1	2
23	Baula	0,460	0,449	2	0.987	0.013	1	131	Pasikolaga	0,242	0,036	2	0.975	0.025	1
24	Benua	0,806	1,812	1	0.003	0.997	2	132	Pasir Putih	0,174	0,053	2	0.982	0.018	1
25	Besulutu	0,518	0,353	2	0.952	0.048	1	133	Poasia	0,383	4,711	1	0	1	2
26	Betoambari	0,671	1,792	1	0.392	0.608	2	134	Poleang	1,386	1,098	2	0.628	0.372	1
27	Binongko	14,693	2,084	2	0	1	2	135	Poleang Barat	0,478	0,484	1	0.977	0.023	1
28	Bondoala	0,122	0,035	2	0.972	0.028	1	136	Poleang Selatan	0,474	0,344	2	0.944	0.056	1
29	Bone	0,158	0,207	1	0.953	0.047	1	137	Poleang Tengah	0,280	0,091	2	0.98	0.02	1
30	Bonegunu	0,302	0,141	2	0.965	0.035	1	138	Poleang Tenggara	0,502	0,183	2	0.967	0.033	1
31	Buke	0,296	1,113	1	0.797	0.203	1	139	Poleang Timur	0,999	0,499	2	0.716	0.284	1
32	Bungi	0,161	0,029	2	0.983	0.017	1	140	Poleang Utara	0,474	0,499	1	0.933	0.067	1
33	Dangia	0,294	0,241	2	0.974	0.026	1	141	Polia-Polia	1,815	0,179	2	0.975	0.025	1
34	Duruka	0,146	0,376	1	0.962	0.038	1	142	Pomalaa	4,270	8,428	1	0	1	2
35	Gu	0,491	0,700	1	0.983	0.017	1	143	Pondidaha	0,357	0,238	2	0.971	0.029	1
36	Iwoimendaa	0,299	0,325	1	0.949	0.051	1	144	Porehu	0,178	0,066	2	0.975	0.025	1
37	Kabaena	0,170	0,054	2	0.975	0.025	1	145	Puriala	0,132	0,051	2	0.975	0.025	1
38	Kabaena Barat	0,645	0,049	2	0.972	0.028	1	146	Puuwatu	0,385	3,598	1	0.281	0.719	2
39	Kabaena Selatan	0,670	0,090	2	0.973	0.027	1	147	Ranomeeto	1,359	1,809	1	0.01	0.99	2
40	Kabaena Tengah	0,333	0,104	2	0.963	0.037	1	148	Ranomeeto Barat	0,061	0,163	1	0.973	0.027	1
41	Kabaena Timur	0,583	0,654	1	0.569	0.431	1	149	Ranteangin	0,215	0,054	2	0.983	0.017	1
42	Kabaena Utara	0,349	0,123	2	0.962	0.038	1	150	Rarowatu	0,285	0,188	2	0.968	0.032	1
43	Kabangka	1,038	0,314	2	0.758	0.242	1	151	Rarowatu Utara	0,514	0,099	2	0.985	0.015	1
44	Kabawo	0,859	0,107	2	0.88	0.12	1	152	Routa	0,147	0,054	2	0.959	0.041	1
45	Kadatua	1,429	0,448	2	0.898	0.102	1	153	Rumbia	0,984	1,029	1	0.972	0.028	1
46	Kadia	2,610	21,948	1	0	1	2	154	Rumbia Tengah	0,409	0,322	2	0.945	0.055	1
47	Kaledupa	0,713	0,395	2	0.847	0.153	1	155	Sabulakoa	0,098	0,084	2	0.978	0.022	1
48	Kaledupa Selatan	0,160	0,052	2	0.98	0.02	1	156	Samaturu	2,834	1,900	2	0	1	2
49	Kalisusu	2,613	0,728	1	0.017	0.983	2	157	Sampara	0,063	0,200	1	0.942	0.058	1
50	Kalisusu Barat	0,195	0,024	2	0.985	0.015	1	158	Sampolawa	2,986	1,258	2	0	1	2
51	Kalisusu Utara	0,703	0,151	2	0.981	0.019	1	159	Sangia Wambulu	0,267	0,067	2	0.955	0.045	1
52	Kaloka	0,518	3,331	1	0	1	2	160	Sawa	0,160	0,129	2	0.973	0.027	1
53	Kambu	1,808	4,883	1	0	1	2	161	Sawerigadi	0,107	0,053	2	0.964	0.036	1
54	Kapoiala	0,220	0,161	2	0.974	0.026	1	162	Siompu	6,441	1,018	2	0	1	2
55	Kapontori	1,821	0,619	2	0.002	0.998	2	163	Siompu Barat	0,907	0,552	2	0.064	0.936	2
56	Katobu	1,579	5,021	1	0.156	0.844	2	164	Siontapina	7,208	1,380	2	0	1	2
57	Kendari	1,152	4,207	1	0	1	2	165	Sorawolio	0,200	0,166	2	0.929	0.071	1
58	Kendari Barat	0,914	8,920	1	0	1	2	166	Soropia	0,136	0,112	2	0.982	0.018	1
59	Kodeoha	0,266	0,220	2	0.976	0.024	1	167	Talaga Raya	0,627	0,937	1	0.961	0.039	1
60	Kokalukuna	0,069	0,262	1	0.978	0.022	1	168	Tanggetada	0,075	0,520	1	0.967	0.033	1
61	Kolono	0,300	1,395	1	0.869	0.131	1	169	Taori	0,574	1,411	1	0.356	0.644	2
62	Kombawa	0,244	0,196	2	0.973	0.027	1	170	Tinanggea	0,661	2,409	1	0.439	0.561	2
63	Konda	0,918	3,204	1	0.081	0.919	2	171	Tinondo	0,064	0,130	1	0.974	0.026	1
64	Konowe	0,216	0,172	2	0.961	0.039	1	172	Tirawuta	0,338	0,485	1	0.817	0.183	1
65	Kontukowuna	0,308	0,120	2	0.982	0.018	1	173	Tiworo Kepulauan	0,301	0,171	2	0.98	0.02	1
66	Kontunaga	0,170	0,152	2	0.982	0.018	1	174	Tiworo Selatan	0,212	0,179	2	0.973	0.027	1
67	Kusambi	0,288	0,331	1	0.933	0.067	1	175	Tiworo Tengah	0,315	0,135	2	0.968	0.032	1
68	Ladongi	0,344	0,690	1	0.983	0.017	1	176	Tiworo Utara	0,273	0,194	2	0.933	0.067	1
69	Laeya	0,197	1,529	1	0.72	0.28	1	177	Tiwu	0,245	0,065	2	0.979	0.021	1
70	Lainea	0,112	0,203	1	0.971	0.029	1	178	Tomia	0,360	0,058	2	0.958	0.042	1
71	Lakudo	6,108	2,460	2	0	1	2	179	Tomia Timur	0,279	0,089	2	0.984	0.016	1
72	Lalembuu	0,048	0,195	1	0.986	0.014	1	180	Tongauna	0,093	0,227	1	0.946	0.054	1
73	Lalolae	0,074	0,037	2	0.983	0.017	1	181	Tongauna Utara	0,103	0,144	1	0.965	0.035	1
74	Lalonggasumeeto	0,178	0,202	1	0.953	0.047	1	182	Tongkuno	0,269	0,486	1	0.98	0.02	1
75	Lambadia	0,271	0,725	1	0.969	0.031	1	183	Tongkuno Selatan	0,384	0,083	2	0.955	0.045	1
76	Lambai	0,219	0,140	2	0.988	0.012	1	184	Tontonunu	0,438	0,107	2	0.979	0.021	1
77	Lambuya	0,161	0,190	1	0.951	0.049	1	185	Towea	1,489	0,141	2	0.882	0.118	1
78	Landono	0,043	0,116	1	0.982	0.018	1	186	Uepai	0,200	0,155	2	0.969	0.031	1
79	Langgikima	0,187	0,046	2	0.961	0.039	1	187	Uesi	0,147	0,017	2	0.982	0.018	1
80	Lantari Jaya	0,683	0,121	2	0.981	0.019	1	188	Uluiwoi	0,247	0,071	2	0.976	0.024	1
81	Laonti	0,034	0,134	1	0.933	0.067	1	189	Unaaha	0,360	1,036	1	0.002	0.998	2
82	Lapandewa	1,006	0,326	2	0.34	0.66	2	190	Wabula	1,240	0,101	2	0.725	0.275	1
83	Lasalepa	0,412	0,029	2	0.982	0.018	1	191	Wadaga	0,242	0,064	2	0.976	0.024	1
84	Lasalimu	1,368	0,621	2	0.002	0.998	2	192	Wakorumba	0,396	0,224	2	0.983	0.017	1
85	Lasalimu Selatan	1,258	0,901	2	0.08	0.92	2	193	Wakorumba Selatan	0,292	0,027	2	0.972	0.028	1
86	Lasolo	0,352	0,093	2	0.966	0.034	1	194	Wangi-Wangi	7,056	1,322	2	0.614	0.386	1
87	Lasolo Kepulauan	0,131	0,049	2	0.967	0.033	1	195	Wangi-Wangi Selatan	7,361	1,883	2	0	1	2
88	Lasusua	1,730	6,367	1	0	1	2	196	Watopute	1,422	0,222	2	0.885	0.115	1
89	Latambaga	1,406	4,884	1	0.002	0.998	2	197	Watubangga	0,899	1,946	1	0.147	0.853	2
90	Latoma	0,138	0,044	2	0.966	0.034	1	198	Watunohu	0,255	0,011	2	0.984	0.016	1
91	Lawa	0,237	0,055	2	0.979	0.021	1	199	Wawo	0,124	0,025	2	0.979	0.021	1
92	Lea-Lea	0,278	0,034	2	0.972	0.028	1	200	Wawonii Barat	0,276	0,085	2	0.962	0.038	1
93	Lembo	0,156	0,066	2	0.969	0.031	1	201	Wawonii Selatan	1,986	0,176	2	0.95	0.05	1
94	Loea	0,114	0,040	2	0.986	0.014	1	202	Wawonii Tengah	0,259	0,046	2	0.976	0.024	1
95	Lohia	1,341	0,196	2	0.816	0.184	1	203	Wawonii Tenggara	0,350	0,045	2	0.975	0.025	1
96	Maginti	0,226	0,154	2	0.986	0.014	1	204	Wawonii Timur	0,108	0,029	2	0.978	0.022	1
97	Maligano	0,263	0,063	2	0.981	0.019	1	205	Wawonii Timur Laut	0,304	0,028	2	0.973	0.027	1
98	Mandonga	0,202	0,847	1	0.759	0.241	1	206	Wawonii Utara	0,063	0,037	2	0.986	0.014	1
99	Marobo	0,108	0,217	1	0.978	0.022	1	207	Wawotobi	0,246	0,437	1	0.941	0.059	1
100	Maroma Utara	0,040	0,196	1	0.964	0.036	1	208	Wiwirano	0,357	0,122	2	0.957	0.043	1
101	Masaloka Raya	0,217	0,086	2	0.975	0.025	1	209	Wolasi	0,114	0,194	1	0.98	0.02	1
102	Mataoleo	0,888	0,079	2	0.977	0.023	1	210	Wolio	1,603	9,259	1	0	1	2
103	Matausu	0,273	0,103	2	0.974	0.026	1	211	Wolowa	0,296	0,016	2	0.982	0.018	1
104	Mawasangka	5,847	3,987	2	0	1	2	212	Wolowa	0,571	1,881	1	0.067	0.933	2
105	Mawasangka Tengah	1,288	0,316	2	0.116	0.884	2	213	Wonggeduku	0,113	0,288	1	0.909	0.091	1
106	Mawasangka Tengah	0,208	0,041	2	0.979	0.021	1	214	Wonggeduku Barat	2,410	0,217	2	0.776	0.224	1
107	Mawasangka Timur	0,548	0,156	2	0.962	0.038	1	215	Wua-Wua	0,365	2,565	1	0	1	2
108	Meluhu	0,232	0,179	2	0.916	0.084	1	216	Wundulako	2,587	1,292	2	0.001	0.999	2

## Data Availability

The authors do not have permission to share data.
